# Profile of Antiemetic Activity of Netupitant Alone or in Combination with Palonosetron and Dexamethasone in *Ferrets* and *Suncus murinus* (House Musk Shrew)

**DOI:** 10.3389/fphar.2016.00263

**Published:** 2016-08-31

**Authors:** John A. Rudd, Man P. Ngan, Zengbing Lu, Guy A. Higgins, Claudio Giuliano, Emanuela Lovati, Claudio Pietra

**Affiliations:** ^1^Emesis Research Group, School of Biomedical Sciences, Faculty of Medicine, The Chinese University of Hong KongHong Kong, China; ^2^Brain and Mind Institute, The Chinese University of Hong KongHong Kong, China; ^3^Intervivo Solutions Inc., TorontoON, Canada; ^4^Research and Preclinical, Helsinn Healthcare SA., LuganoSwitzerland

**Keywords:** anti-emetic, nausea, vomiting, chemotherapy, 5-HT_3_, NK_1_, ferret

## Abstract

**Background and Aims:** Chemotherapy-induced acute and delayed emesis involves the activation of multiple pathways, with 5-hydroxytryptamine (5-HT; serotonin) playing a major role in the initial response. Substance P tachykinin NK_1_ receptor antagonists can reduce emesis induced by disparate emetic challenges and therefore have a clinical utility as broad inhibitory anti-emetic drugs. In the present studies, we investigate the broad inhibitory anti-emetic profile of a relatively new NK_1_ receptor antagonist, netupitant, alone or in combination with the long acting 5-HT_3_ receptor antagonist, palonosetron, for a potential to reduce emesis in ferrets and shrews.

**Materials and Methods:** Ferrets were pretreated with netupitant and/or palonosetron, and then administered apomorphine (0.125 mg/kg, s.c.), morphine (0.5 mg/kg, s.c.), ipecacuanha (1.2 mg/kg, p.o.), copper sulfate (100 mg/kg, intragastric), or cisplatin (5–10 mg/kg, i.p.); in other studies netupitant was administered to *Suncus murinus* before motion (4 cm horizontal displacement, 2 Hz for 10 min).

**Results:** Netupitant (3 mg/kg, p.o.) abolished apomorphine-, morphine-, ipecacuanha- and copper sulfate-induced emesis. Lower doses of netupitant (0.03–0.3 mg/kg, p.o.) dose-dependently reduced cisplatin (10 mg/kg, i.p.)-induced emesis in an acute (8 h) model, and motion-induced emesis in *S. murinus*. In a ferret cisplatin (5 mg/kg, i.p.)-induced acute and delayed emesis model, netupitant administered once at 3 mg/kg, p.o., abolished the first 24 h response and reduced the 24–72 h response by 94.6%; the reduction was markedly superior to the effect of a three times per day administration of ondansetron (1 mg/kg, i.p.). A single administration of netupitant (1 mg/kg, p.o.) plus palonosetron (0.1 mg/kg, p.o.) combined with dexamethasone (1 mg/kg, i.p., once per day), also significantly antagonized cisplatin-induced acute and delayed emesis and was comparable with a once-daily regimen of ondansetron (1 mg/kg, p.o.) plus aprepitant (1 mg/kg, p.o.) in combination with dexamethasone (1 mg/kg, i.p.).

**Conclusion:** In conclusion, netupitant has potent and long lasting anti-emetic activity against a number of emetic challenges indicating broad inhibitory properties. The convenience of protection afforded by the single dosing of netupitant together with palonosetron was demonstrated and also is known to provide an advantage over other therapeutic strategies to control emesis in man.

## Introduction

The treatment of cancer with chemotherapeutic agents such as cisplatin is documented to be associated with a number of side effects including nausea and emesis, which can be reduced by agents blocking 5-HT_3_ and substance P NK_1_ receptors (Rudd and Andrews, 2004; Hesketh, 2008). It has been hypothesized that there is an initial release of 5-HT (serotonin) from enterochromaffin cells in the gastrointestinal tract to activate 5-HT_3_ receptors located on vagal afferents (Naylor and Rudd, 1996; Minami et al., 2003). The mechanism of release is not entirely known but may involve free radical generation and/or cellular damage, which subsequently leads to the involvement of other neurotransmitter systems and/or mediators (Andrews and Rudd, 2015). The response occurring over the first 0–24 h has become known as the acute response, and ‘first generation’ 5-HT_3_ receptor antagonists, such as ondansetron and granisetron, have been used widely to reduce nausea and emesis during this phase (Hesketh, 2008). However, 5-HT_3_ receptor antagonists are less effective to control nausea and emesis occurring during the post 24 h period, which became known as the delayed phase of emesis (Rudd and Andrews, 2004).

To improve the overall control of acute and delayed emesis, 5-HT_3_ receptor antagonists were initially combined with glucocorticoids such as dexamethasone (Hesketh et al., 1994; Ioannidis et al., 2000). Subsequently, preclinical studies identified that brain penetrating tachykinin NK_1_ receptor antagonists had a broad inhibitory profile to inhibit emesis induced by disparate challenges (Bountra et al., 1993; Tattersall et al., 1993, 1994; Andrews and Rudd, 2004). It was also shown that NK_1_ receptor antagonists had anti-emetic activity in a ferret model of acute and delayed emesis prompting clinical testing (Rudd et al., 1996; Singh et al., 1997; Tattersall et al., 2000; Tsuchiya et al., 2002). The standard regimen to control both phases of emesis in man quickly changed to a triple regimen of a 5-HT_3_ receptor antagonist, in combination with the first licensed NK_1_ receptor antagonist for chemotherapy-induced emesis, aprepitant, plus a glucocorticoid (Jordan et al., 2005; Roila et al., 2010; Bayo et al., 2012). Unfortunately, however, even with these advances, there still remains a proportion of patients not adequately protected from chemotherapy-induced nausea and emesis (Navari, 2004; Aranda Aguilar et al., 2005).

Palonosetron is a ‘second generation’ 5-HT_3_ receptor antagonist that is an order of magnitude more potent than older compounds at blocking 5-HT_3_ receptors and has excellent bioavailability following oral administration (F 99%); it also has a plasma half-life that is approximately three times longer, enabling a convenient once per day administration (Wong et al., 1995; Grunberg and Koeller, 2003; De Leon, 2006). In the clinical setting, palonosetron was shown to be superior to the first generation 5-HT_3_ receptor antagonists, particularly in its ability to reduce delayed nausea and emesis (Rubenstein, 2004; Geling and Eichler, 2005; Tonini et al., 2005). It was speculated that the unique profile of palonosetron may relate to its additional ability to prevent 5-HT_3_ receptor re-cycling and/or a potential to reduce substance P responses mediated via NK_1_ receptors by preventing 5-HT_3_ and NK_1_ receptor cross-talk (Rojas et al., 2008, 2010b, 2014; Stathis et al., 2012).

Since the clinical introduction of aprepitant, there have also been advances in the design of more potent and longer acting tachykinin NK_1_ receptor antagonists (Reddy et al., 2006; Rojas et al., 2014). Netupitant is a novel orally active compound that penetrates into the brain and has a long duration of action and an insurmountable blocking activity at NK_1_ receptors (Rizzi et al., 2012). Studies using NG108-15 cells have shown that netupitant and palonosetron have synergistic effects to antagonize substance P-induced calcium mobilization; synergism was not seen when netupitant was combined with ondansetron or granisetron (Stathis et al., 2012). Clinically, netupitant plus dexamethasone was shown to be superior to palonosetron plus dexamethasone against moderately emetogenic chemotherapy (Aapro et al., 2014), but the combination of netupitant and palonosetron with dexamethasone provided an excellent control of highly emetogenic chemotherapy induced-acute and delayed emesis, showing improvements over ondansetron and aprepitant combinations, with efficacy being maintained over multiple cycles (Gralla et al., 2014; Hesketh et al., 2014; Navari, 2015).

In the present studies, we used the ferret, a species with proven translational value in anti-emetic research (Percie du Sert et al., 2011), to explore the potential of a single administration of netupitant alone or in combination with palonosetron to inhibit cisplatin-induced acute and delayed emesis following an oral administration, compared with the control of emesis afforded by the three times per day administration of ondansetron alone, or when ondansetron was used daily combined with aprepitant and dexamethasone (Tattersall et al., 2000). An attempt was also made to characterize the spectrum of anti-emetic activity of netupitant to reduce emesis induced by other challenges. Apomorphine and morphine were selected to induce emesis via the area postrema (Lau et al., 2005; Percie du Sert et al., 2009), and intragastric copper sulfate was chosen to induce emesis via peripheral vagal and splanchnic pathways from the gastrointestinal tract (Kan et al., 2006). Ipecacuanha was selected as an emetogen inducing emesis via mixed central and peripheral pathways (Ariumi et al., 2000; Hasegawa et al., 2002). We also utilized *Suncus murinus* (house musk shrew) to investigate the potential of netupitant to reduce provocative motion-induced emesis that involves central pathways via vestibular inputs (Ueno et al., 1988; Ito et al., 2003). The studies described represent a reference point for others to compare the anti-emetic activity of new chemical entities or therapeutic strategies for the treatment of emesis.

## Materials and Methods

### Animals

Male castrated ferrets (0.8–1.8 kg) were obtained from the University of Leeds (England) or Southland Ferrets (Invercargill, New Zealand). Water and food (SDS Diet ‘C,’ Special Diet Services, Ltd., UK, or TriPro super premium chicken meal formula dog food, American Nutrition, USA) were given *ad libitum* unless otherwise stated. Male *S. murinus* (45–65 g) were obtained from the University of Bradford (Bradford, U.K.). Dry pelleted trout pellets (Aquatic 3, Special Diet Services, UK) and water were given *ad libitum* unless otherwise stated. Animals were housed in temperature-controlled rooms at 20–24 ± 1°C under artificial lighting, with lights on between 06:00 and 18:00 h. The relative humidity was maintained at 50 ± 5%. All experiments were conducted under license from the respective Governments of England, Switzerland and Hong Kong, and were approved by the relevant Animal Experimentation Ethics committees.

### Determination of the Plasma Half-Life of Netupitant in Ferrets

Ferrets were anesthetized with halothane (3%) in oxygen and the left jugular vein was cannulated and exteriorized to the back of the neck using standard surgical techniques (Barnes et al., 1991). Animals were then allowed to recover for 2 weeks. On the day of the experiment, at *t* = 0, a 1.5 ml blood sample was withdrawn before administering netupitant (3 mg/kg, p.o.). Subsequently, further blood sampling (1.5 ml) was continued at 1, 3, 6, 12, 24, 36, 48, 60, 72, 84, and 96 h. Each sample was centrifuged at 10,000 *g* for 10 min and the plasma was stored at -80°C prior to analysis. The analytical method used to determine the plasma concentration of netupitant was essentially the same as described by Huskey et al. (2003).

### Experiments in Ferrets Involving Apomorphine, Morphine, Ipecacuanha, and Cisplatin during Acute Observation Times

On the day of experiment, the animals were transferred to individual cages where they were allowed at least 30 min to adapt before being presented with approximately 100 g of food. Netupitant (0.03–3 mg/kg) or vehicle [0.3% (v/v) Tween 80 in saline; 2 ml/kg] was then administered orally 2 h before the subcutaneous administration of apomorphine (0.125 mg/kg, s.c.), morphine (0.5 mg/kg, s.c.), ipecacuanha (1.2 mg/kg, p.o.), copper sulfate (100 mg/kg, i.g.), or cisplatin (10 mg/kg, i.p.). Animal behavior was recorded for up to 8 h using a closed circuit video recording system.

### Experiments Involving *Suncus murinus* and Provocative Motion

The protocol used for the provocative motion-induced emesis experiments have been described previously (Chan et al., 2007). Briefly, the animals were transferred to the behavioral laboratory and given 30 min to habituate before being administered netupitant (0.03–3 mg/kg) or vehicle [0.3% (v/v) Tween 80 in saline; 2 ml/kg] orally. After 105 min, they were transferred to individual chambers (21 cm × 14 cm × 13 cm) and given 15 min to adapt before starting the provocative motion stimulus (i.e., 120 min post netupitant/vehicle administration; 4 cm horizontal displacement, 2 Hz, using a Heidolph Promax 2020 desktop shaker, Labplant, England). Animal behavior was recorded for 10 min using a closed circuit video recording system (Chan et al., 2007).

### Experiments to Assess Anti-Emetic Potency on Cisplatin-Induced Acute and Delayed Emesis

The ferret acute and delayed emesis model has been described previously (Rudd and Naylor, 1996). Animals were transferred to individual observation cages where they were allowed at least 48 h to adapt. A dry pellet diet (Laboratory Feline Diet 5003, PMI Nutrition Inc., St. Louis, MO, USA) and water was available *ad libitum*. Animals were presented with 100 g of commercially available cat food (Whiskas^®^, Effem Foods Pty. Ltd., Wodonga, Australia) 30 min prior to drug/vehicle administration. In an initial series of experiments, ferrets were administered with cisplatin (5 mg/kg) intraperitoneally followed immediately by the oral administration of vehicle [0.3% (v/v) Tween 80 in saline; 2 ml/kg], netupitant (1–3 mg/kg), or ondansetron (1 mg/kg); the administration of vehicle or ondansetron was repeated at 8 h intervals.

In other experiments, ferrets were allowed a 2-day habituation period. Some ferrets were administered ondansetron (1 mg/kg, p.o.), or netupitant (3 mg/kg, p.o.), 2 h prior to the administration of cisplatin (5 mg/kg, i.p.); the administration of ondansetron was repeated at 8 h intervals. In other experiments, ferrets were administered palonosetron (0.1 mg/kg, p.o.) and/or netupitant (1 mg/kg, p.o.), in combination with dexamethasone (1 mg/kg, i.p.), or respective vehicles (2 ml/kg) 15 min before cisplatin (5 mg/kg, i.p); dexamethasone (1 mg/kg, i.p.) or vehicle (distilled water, 1 ml/kg) was administered 15 min before cisplatin (5 mg/kg, i.p.) and then repeated at 24 h intervals. Some animals were also randomized as positive controls and administered ondansetron (1 mg/kg, p.o.) plus aprepitant (1 mg/kg, p.o.) orally 15 min before cisplatin and dexamethasone (1 mg/kg, i.p.) 15 min before cisplatin; ondansetron, and dexamethasone administrations were repeated at 24 h intervals. Respective vehicle controls (distilled water, 2 ml/kg) were also utilized.

### Measurement of Emesis

Emesis was characterized by rhythmic abdominal contractions that were either associated with the forceful oral expulsion of solid or liquid material from the gastrointestinal tract (i.e., vomiting), or not associated with the passage of material (i.e., retching movements). For ferrets, consecutive episodes of retching and/or vomiting were considered separate when the animal changed its location in the observation cage, or when the interval between episodes exceeded 5 s (Rudd and Naylor, 1996). For *S. murinus*, episodes of emesis were also characterized as described above, except that the interval to demarcate two consecutive episodes of retching and/or vomiting was 2 s (Rudd et al., 1999). At the end of the observation period, animals were terminated by an intraperitoneal injection of pentobarbitone sodium (80 mg/kg).

### Data Analysis

Data are expressed as the mean ± SEM, unless otherwise stated. Half-life values and ID_50_ values were calculated from data expressed as a percentage of the control response using linear and non-linear regression analysis, respectively. In all cases, differences between treatment groups were considered significant when *P* < 0.05 (Student’s *T*-test, or one way ANOVA and Tukey’s multiple comparison tests, as appropriate; GraphPad Prism version 5.0, Inc. Version, San Diego, CA, USA).

### Drug Formulation

Ondansetron hydrochloride d-hydrate, dexamethasone 21-phosphate disodium salt, and D-mannitol were from Sigma–Aldrich, St. Louis, MO, USA. Cisplatin (1 mg/ml in 0.1% mannitol in saline) was from David Bull Laboratories, Mulgrave, VIC, Australia. Palonosetron hydrochloride, aprepitant and netupitant were from Helsinn Advanced Synthesis SA, Switzerland. Ondansetron, dexamethasone, and palonosetron were dissolved in distilled water. Netupitant was dissolved in 0.3% Tween 80 in Saline (0.9% w/v). Aprepitant was dissolved in a solution of ethanol:propylene glycol:distilled water in the ratio of 1:6:3. Doses are expressed as the free base and dosing volumes were 1 ml/kg.

## Results

### Pharmacokinetic Profile of Netupitant in Ferrets

The plasma level of netupitant peaked 1–3 h following its oral administration; the *T*_max_ was 2.5 ± 0.5 h and the *C*_max_ was 397.3 ± 19.7 ng/ml (*n* = 4). The plasma level of netupitant was measurable up to 96 h and had a flat terminal profile with a terminal half-life of 78.5 ± 17.0 h (*n* = 4).

### Anti-Emetic of Activity of Netupitant against Drug- and Motion-Induced Emesis in the Ferret

**FIGURE 1:**
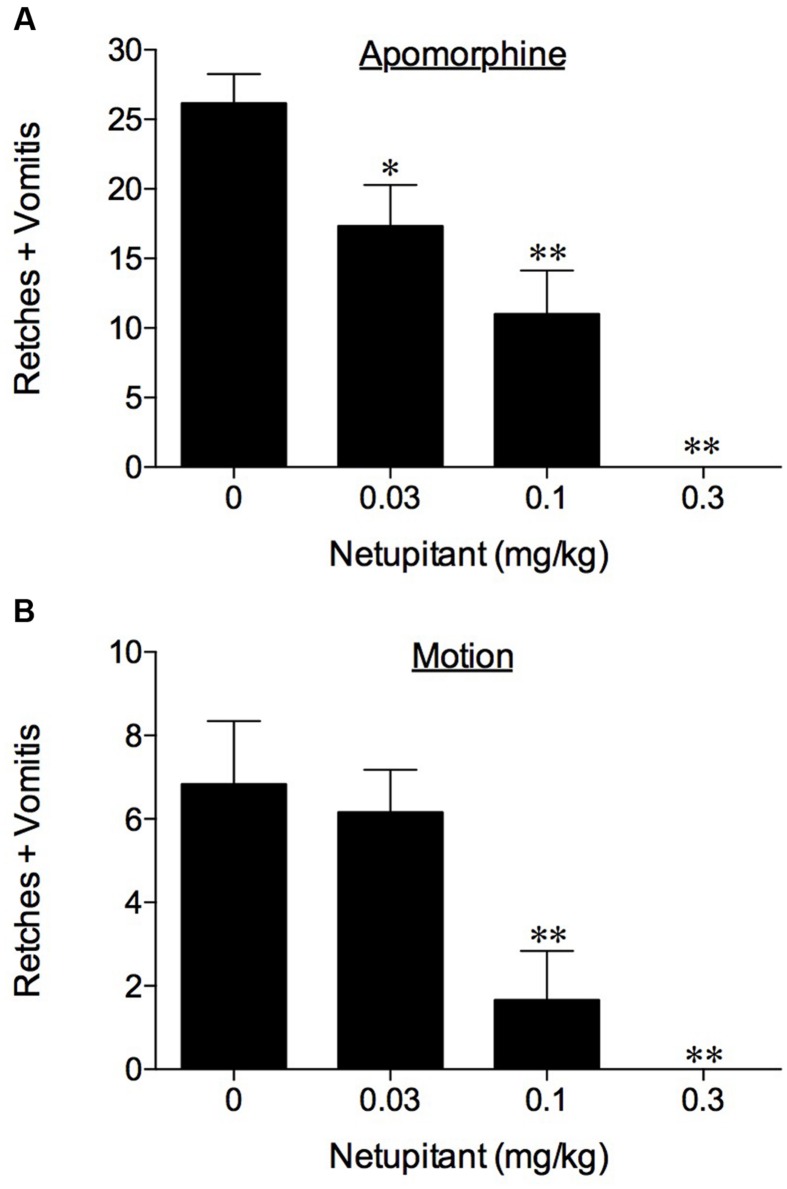
**The effect of netupitant on (A) apomorphine (0.125 mg/kg, s.c.)-induced emesis in ferrets and (B) motion-induced emesis in *Suncus murinus*.** Netupitant (0.03–0.3 mg/kg) or vehicle was administered orally 2 h prior to apomorphine (0.125 mg/kg, s.c.) or motion (4 cm horizontal displacement, 2 Hz for 10 min). Significant differences relative to vehicle treated animals (0 mg/kg) are indicated as ^∗^*P* < 0.05 or ^∗∗^*P* < 0.01 (one way ANOVA followed by Tukey’s multiple comparison testing).

No retching or vomiting was observed during the habituation periods and there were no obvious differences in the behavior of the animals randomized to the treatment groups prior to drug/vehicle administration. Netupitant was not associated with emesis during the pre-treatment period. Apomorphine, morphine and copper sulfate subsequently induced emesis in the vehicle treated animals following a short latency of ~ 5 min (data not shown). The range of retches + vomits recorded during the 30 min observation periods were: apomorphine, 21–35; morphine, 33–55; and copper sulfate, 60–100. The oral administration of netupitant at 3 mg/kg completely prevented the retching and vomiting response to these challenges (*P* < 0.01; **Table 1**). A more detailed dose-ranging experiment against apomorphine (control retches + vomits range: 17–32) revealed that a significant 33.8% reduction of retching + vomiting could be observed at doses of netupitant as low as 0.03 mg/kg (*P* < 0.05); increasing the doses to 0.1 mg/kg reduced emesis by 75.6% (*P* < 0.01) and 0.3 mg/kg prevented emesis completely (*P* < 0.01; **Figure 1**). The ID_50_ to inhibit apomorphine-induced retching + vomiting was 0.08 mg/kg, p.o. In the experiments involving ipecacuanha, the vehicle treated control animals exhibited emesis following a latency period of approximately 20 min (data not shown) that comprised ~38 retches + vomits during the 60 min observation period. The emetic response induced by ipecacuanha was similarly prevented completely by netupitant at 3 mg/kg, p.o. (*P* < 0.05; **Table 1**). No dose-ranging experiments were done against ipecacuanha, copper sulfate or morphine, so ID_50_ values could not be calculated.

**Table 1:** Ability of netupitant to abolish retching and vomiting induced by apomorphine, morphine, ipecacuanha, and copper sulfate pentahydrate.

Treatment	Apomorphine (0.125 mg/kg, s.c.)	Morphine (0.5 mg/kg, s.c.)	Ipecacuanha (1.2 mg/kg, p.o.)	Copper Sulphate (100 mg/kg, intragastric)
Vehicle	26.2 ± 2.8	43.8 ± 3.7	37.5 ± 6.8	78.8 ± 6.5
Netupitant 3 mg/kg, p.o.	0.0 ± 0.0^∗∗^	0.0 ± 0.0^∗∗^	0.0 ± 0.0^∗∗^	0.0 ± 0.0^∗∗^

*Netupitant (3 mg/kg) or vehicle, was administered orally 2 h prior to challenge with emetic drugs. Data represents the mean ± SEM of the number of retches + vomits recorded during a 30–60 min observation period. Significant differences relative to the vehicle control treated animals are indicated as ^∗∗^P < 0.01 (Student’s unpaired t-test; n = 6).*

**FIGURE 2:**
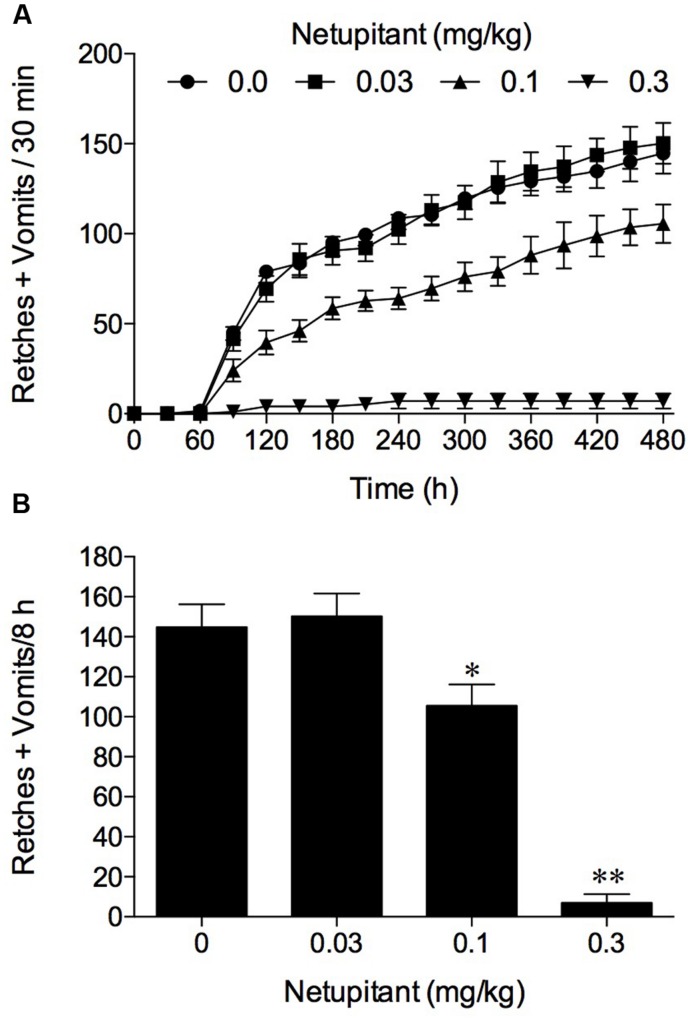
**The effect of netupitant on cisplatin (10 mg/kg, i.p.)-induced emesis in ferret.** Netupitant (0.03–0.3 mg/kg) or vehicle was administered orally 2 h prior to cisplatin (10 mg/kg, i.p.; *t* = 0). Data represents the mean ± SEM of **(A)** cumulative numbers of retches + vomits per 30 min, or **(B)** the total numbers of retches + vomits occurring over 8 h (*n* = 5–6). Significant differences relative to vehicle treated animals (0 mg/kg) are indicated as ^∗^*P* < 0.05, ^∗∗^*P* < 0.01 (one way ANOVA followed by Tukey’s multiple comparison testing).

In the experiments involving the use of cisplatin at 10 mg/kg, i.p., the vehicle treated control animals exhibited emesis after 60–90 min that comprised ~145 retches + vomits (range: 103–173) during the 8 h observation period. Netupitant at 0.1 mg/kg antagonized significantly the retching + vomiting response by 27.1% (*P* < 0.05) and increasing the dose of netupitant to 0.3 mg/kg, p.o., antagonized the response by 95.2% (**Figure 2**; *P* < 0.01); the ID_50_ to inhibit emesis was approximately 0.1 mg/kg, p.o.

### Anti-Emetic of Activity of Netupitant against Provocative Motion (4 cm Horizontal Displacement at 2 Hz for 10 min)-Induced Emesis

In the experiments involving provocative motion, five out of six vehicle treated control animals exhibited emesis after a short latency (~2–4 min) that comprised ~8 retches + vomits (range: 7–11) during the 10 min observation period. Netupitant at 0.1 mg/kg, p.o. antagonized significantly the retching + vomiting response by 75.6% (*P* < 0.01), and increasing the dose of netupitant to 0.3 mg/kg, p.o., prevented the response completely (**Figure 1**; *P* < 0.01); the ID_50_ to inhibit emesis was 0.08 mg/kg, p.o.

### Anti-Emetic Potential of Netupitant Compared with Ondansetron to Inhibit Cisplatin (5 mg/kg, i.p.)-Induced Acute and Delayed Emesis

**FIGURE 3:**
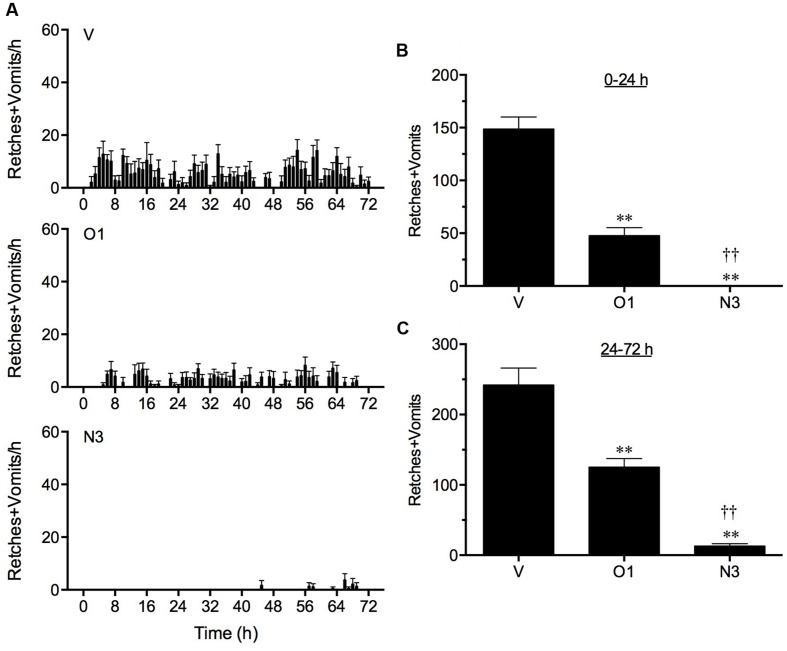
**The effect of ondansetron (O1) or netupitant (N3) on the profile of retching and/or vomiting induced by cisplatin in the ferret.** Ondansetron, (1 mg/kg, p.o.), was administered 15 min before cisplatin (5 mg/kg, i.p.; *t* = 0) and then at regular 8 h intervals; netupitant (3 mg/kg, p.o.) was administered once, 2 h prior to cisplatin. Data represents the mean ± SEM of the total numbers of retches + vomits occurring in **(A)** 1, **(B)** 0–24, or **(C)** 24–72 h time intervals, as appropriate (*n* = 5–6). Significant differences relative to vehicle treated animals (V) are indicated as ^∗∗^*P* < 0.01; significant differences relative to ondansetron treated animals are indicated as ^††^*P* < 0.01 (one way ANOVA followed by Tukey’s multiple comparison testing).

In vehicle treated animals, cisplatin induced 148.7 ± 11.5 and 242.2 ± 24.0 retches + vomits during the acute (0–24 h) and delayed (24–72 h) periods, respectively (**Figure 3**). The three times per day administration of ondansetron, 1 mg/kg, p.o., reduced the acute response significantly by 67.8% (*P* < 0.01) and also reduced the delayed response by 48.3% (*P* < 0.01; **Figure 3**).

Netupitant at 3 mg/kg, p.o., administered 2 h prior to the injection of cisplatin, prevented completely the acute response (*P* < 0.05) and reduced the delayed response significantly by 94.6% (*P* < 0.01; **Figure 3**). In fact, the single administration of netupitant was almost twice as effective as the three times administration of ondansetron to prevent the acute and delayed retching and vomiting response (*P* < 0.01; **Figure 3**).

### Comparison of the Anti-Emetic Activity of the Once Only Administration of Palonosetron and Netupitant with the Standard Regimen of Ondansetron and Aprepitant

**FIGURE 4:**
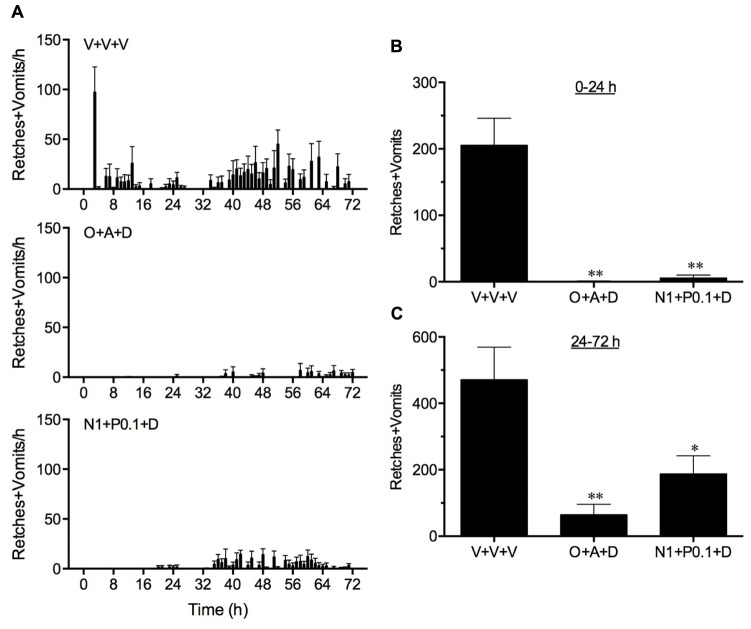
**A comparison of the effect of ondansetron plus aprepitant in combination with dexamethasone (O+A+D) with netupitant plus palonosetron in combination with dexamethasone (N1+P0.1+D) on cisplatin-induced retching and/or vomiting.** Ondansetron, (1 mg/kg, p.o.), aprepitant (1 mg/kg, p.o.), palonosetron (0.1 mg/kg, p.o.), netupitant (1 mg/kg, p.o.), or dexamethasone (1 mg/kg, i.p.), or vehicle, were administered 15 min before cisplatin (5 mg/kg, i.p.; *t* = 0); the administration of ondansetron (1 mg/kg, p.o.) or dexamethasone (1 mg/kg, i.p.) or vehicle was repeated at 24 h intervals. Data represents the mean ± SEM of the total numbers of retches + vomits occurring in **(A)** 1, **(B)** 0–24, or **(C)** 24–72 h time intervals, as appropriate (*n* = 5–6). Significant differences relative to vehicle treated animals (V+V+V) are indicated as ^∗^*P* < 0.05, or ^∗∗^*P* < 0.01 (one way ANOVA followed by Tukey’s multiple comparison testing).

In vehicle treated animals, cisplatin induced 205.6 ± 40.5 and 471.0 ± 98.3 retches + vomits during the acute (0–24 h) and delayed (24–72 h) periods, respectively (**Figure 4**). Both the standard regimen of ondansetron (1 mg/kg, p.o., every 24 h) plus aprepitant (1 mg/kg, p.o., administered once) and dexamethasone (1 mg/kg, i.p., every 24 h), and the single administration of netupitant (1 mg/kg, p.o.) plus palonosetron (0.1 mg/kg, p.o.) in combination with daily dexamethasone (1 mg/kg, i.p.) were highly effective to reduce the acute response significantly by 99.7 (*P* < 0.01) and 97.3% (*P* < 0.01), respectively (**Figure 4**). The standard regimen of ondansetron plus aprepitant and dexamethasone also reduced significantly the delayed emetic response by 86.3% (*P* < 0.01). The netupitant plus palonosetron and dexamethasone regimen reduced delayed emesis to a lesser extent by 60.2% (*P* < 0.05). However, there were no significant differences in the control of acute or delayed emesis by the two anti-emetic regimens (*P* > 0.05).

## Discussion

Netupitant was orally active to antagonize emesis induced by diverse emetogenic stimuli in ferrets and *S. murinus*. This profile is consistent with other NK_1_ receptor antagonists that are presumed to be capable of penetrating the blood brain barrier to reach sites in the dorsal vagal complex and/or sites thought to be adjacent to the semi-compact part of the nucleus ambiguous (Tattersall et al., 1996; Fukuda et al., 1999; Andrews and Rudd, 2004). This is particularly relevant for centrally acting emetogens, such as apomorphine and morphine, or motion, but for other stimuli that may also cause a release of substance P in the periphery, the mechanism may in part also involve NK_1_ receptors on the vagus or perhaps those located elsewhere in the periphery (Minami et al., 1998; Darmani et al., 2008; Ray et al., 2009).

The broad inhibitory anti-emetic profile in ferrets encompassed five challenges where acute emesis was induced: apomorphine [mechanism involving D_2_ receptors at the level of the area postrema; (Lau et al., 2005)], morphine [emetic mechanism involving opioid receptors at the level of the area postrema; (Rudd and Naylor, 1995; Percie du Sert et al., 2009)], copper sulfate [mechanism predominantly involving gastric irritation and vagal and/or splanchnic nerves, but where the transmitter systems are not completely characterized; (Makale and King, 1992; Kan et al., 2006)], ipecacuanha [mechanism involving 5-HT_3_ receptors and vagal and/or splanchnic nerves; (Ariumi et al., 2000; Hasegawa et al., 2002)] and cisplatin [high-dose model – mechanism involving a release of 5-HT from enterochromaffin cells, 5-HT_3_ receptors and vagal and/or splanchnic nerves and activation of the area postrema; (Percie du Sert et al., 2009)]. Several doses of netupitant were utilized to compare potency to inhibit apomorphine- and cisplatin-induced emesis yielding ID_50_ values of ~0.1 mg/kg, p.o., which is five times below its reported potency in the gerbil foot tapping assay [ID_50_ = 0.5 mg/kg, p.o.; (Rizzi et al., 2012)]; at 0.3 mg/kg netupitant inhibited the emetic responses completely. A remarkably similar relative potency difference of other NK_1_ receptor antagonists in the ferret cisplatin-induced emesis assay and the gerbil foot-tapping assay has been reported following intravenously administered brain penetrating NK_1_ receptor antagonists, GR203040, CP-99,994, and L-742,694 (ID_50_ values in foot taping assay ~0.85 mg/kg; ID_50_ values in the cisplatin assay were ~0.18 mg/kg; Rupniak et al., 1997). The consistency of relative potency of the antagonists between studies may reflect netupitant’s excellent bioavailability following oral administration (present investigations). It is also relevant that netupitant binds with high affinity to human NK_1_ receptors (pKi = 9.0), with 1,000 fold selectivity over other tachykinin and G-protein coupled receptors (Rizzi et al., 2012). Predictably, in our studies, the use of netupitant at 3 mg/kg, p.o., abolished morphine- and ipecacuanha-induced emesis; a similar spectrum of effects has been reported for several other NK_1_ receptor antagonists against these emetic stimuli [for a review, see (Andrews and Rudd, 2004)].

We also explored the anti-emetic potential of netupitant in the ferret cisplatin (low-dose 5 mg/kg)-induced acute and delayed emesis model (Rudd et al., 1994; Rudd and Naylor, 1997; Sam et al., 2001). In the present studies, the single oral administration of netupitant at 3 mg/kg was shown to be far superior to the three times per day administration of ondansetron (1 mg/kg, i.p.) at antagonizing both the acute (netupitant abolished acute emesis; ondansetron only reduced acute emesis by ~68%) and delayed response (netupitant reduced delayed emesis by ~95%; ondansetron reduced delayed emesis by ~49%) to cisplatin; the antagonism afforded by ondansetron is comparable to previously published data in this species (Rudd and Naylor, 1994, 1997; Rudd et al., 1996; Singh et al., 1997; Watanabe et al., 2008). These data demonstrate that netupitant has an exceptionally long duration of action which may relate to its exceptionally long plasma half-life of ~79 h (present studies), whereas the plasma half-life of ondansetron is reported to be ~2.3 h (Minami et al., 1991). If we place this in perspective, the first NK_1_ receptor antagonist tested in the ferret acute and delayed emesis model, CP-99,994, [IC_50_ of 1.9 nM to displace [125I]-Bolton Hunter Substance P from ferret brain membranes (Watson et al., 1995)], had a short plasma half-life of ~1.4 h [data from cat, (Lucot et al., 1997)], and even when administered every 8 h at 10 mg/kg, only reduced cisplatin-induced acute emesis by 34% and reduced delayed emesis by ~87% (Rudd et al., 1996); this was the dose of CP-99,994 that was also shown to prevent apomorphine, morphine, ipecacuanha, cisplatin (high-dose)-induced emesis (Bountra et al., 1993; Tattersall et al., 1993; Zaman et al., 2000). Critically, aprepitant, which has an IC_50_ value of 0.7 nM at ferret NK_1_ receptors (0.1 nM for human NK_1_ receptors), and plasma half-life of 10 h [ferret; (Huskey et al., 2003); plasma half-life in humans, 10–29 h; (Bubalo et al., 2012)], only produces a similar level of inhibition of acute and delayed emesis when orally administered at 16 mg/kg (Tattersall et al., 2000).

Palonosetron (RS 25259-197) was first described as a high affinity 5-HT_3_ receptor antagonist [pKi ~10; Wong et al., 1995)] and was subsequently shown to have a plasma half-life of ~40 h in humans (Siddiqui and Scott, 2004; Stoltz et al., 2004). It has been reported to inhibit cisplatin-induced emesis (10 mg/kg, i.v.; 5 h test) in ferrets and dogs with ID_50_ values of 0.003 and 0.008 mg/kg, respectively; the dose of 0.1 mg/kg, p.o., inhibited emesis by almost 100% in both species (Eglen et al., 1995). Indeed, palonosetron was revealed as being twice as potent as ondansetron to inhibit cisplatin-induced emesis in ferrets following oral administration (Eglen et al., 1995). Clinically, palonosetron is recognized as being superior to other 5-HT_3_ receptor antagonists, particularly against chemotherapy-induced delayed emesis (Navari, 2009; Saito et al., 2009). In fact, the magnitude of difference, particularly in the control of delayed emesis, was unexpected; prompting speculation that palonosetron has properties distinct from other selective 5-HT_3_ receptor antagonists. There is strong evidence that it may have a different mechanism of action to ondansetron at the 5-HT_3_ receptor, including an ability to cause receptor internalization, with continued binding to the internalized receptor to prolong the inhibition of its function (not seen with ondansetron and granisetron) and, more recently, an indirect inhibition of substance P responses, *in vivo* (Rojas et al., 2008, 2010a,b).

In the cisplatin (5 mg/kg, i.p.) acute and delayed emesis model, we compared the anti-emetic potential of a regimen of netupitant and palonosetron (both administered before cisplatin) in combination with dexamethasone (administered before cisplatin and then every 24 h), with a regimen comprising ondansetron (administered before cisplatin and then every 24 h) and aprepitant (administered once before cisplatin) in combination with dexamethasone (administered before cisplatin and then every 24 h). Both treatment regimens were highly effective to antagonize acute emesis and were indistinguishable from each other. Indeed, the anti-emetic effect of the regimen of netupitant and palonosetron in combination with dexamethasone was still evident during the delayed phase of the response, and was not significantly different from the control of emesis seen following the more frequent dosing regimen of ondansetron and aprepitant in combination with dexamethasone. These data compare favorably with previous studies investigating aprepitant, ondansetron, and dexamethasone in ferrets (Tattersall et al., 2000).

Palonosetron has also been studied in the *Cryptotis parva* (least shrew). Across a subcutaneous dose range of 0.1–10 mg/kg, palonosetron dose-dependently antagonized the emesis induced by either the 5-HT_3_ receptor agonist, 2 methyl-5-HT (5 mg/kg), i.p., or the L-type calcium channel opener, FLP 64176 (10 mg/kg, i.p.; Darmani et al., 2014). It is unknown why palonosetron could not completely inhibit 2-methyl-5-HT-induced emesis in this species, but the level of inhibition was similar to that reported for tropisetron against the same stimulus (Darmani, 1998).

Palonosetron administered as a single treatment, is not noted to have a U-shaped dose-response curve in ferrets or man against chemotherapy-induced emesis. However, in the least shrew, palonosetron appears to have a species-specific biphasic anti-emetic action against the initial 2 h of emesis induced by cisplatin (10 mg/kg, i.p.), with a significant reduction at 0.1 and 2.5 mg/kg, s.c; while at 0.5 and 5 mg/kg the protection was lost (Darmani et al., 2014). In a subsequent set of experiments, palonosetron was tested at 0.1 mg/kg, s.c., against the same dose of cisplatin but over an extended observation period of 40 h (Darmani et al., 2015). Palonosetron reduced the emesis occurring during the initial first 16 h of the experiment by 83% compared to vehicle pretreated cisplatin controls. An assessment of emesis during the 27–40 h period also recorded a non-significant reduction of 73% (Darmani et al., 2015). Comparatively, netupitant at a high dose of 5 mg/kg, s.c., non-significantly reduced the emesis occurring during the first 16 h by 70% and abolished emesis during the 27–40 h period (Darmani et al., 2015). When both palonosetron and netupitant were combined together, the emesis occurring during the first 16 h was reduced by approximately 94%, and the reduction of emesis in the 27–40 h period was still significantly reduced (Darmani et al., 2015). Thus, the combination of palonosetron and netupitant resulted in a greater level of protection against both acute and ‘delayed’ phases of cisplatin-induced emesis in the least shrew than either treatment administered alone.

*Suncus murinus* is a more commonly used shrew species to study mechanisms of emesis, but its tachykinin receptor has a distinct pharmacology compared to the human and rodent NK_1_ receptor (Tattersall et al., 1995; Rudd et al., 1999). However, in the present studies, netupitant dose-dependently reduced motion-induced emesis, with a dose of 0.3 mg/kg, p.o., preventing the response completely. In comparison with data in the literature, netupitant appears more potent than other NK_1_ receptor antagonists including CP-99,994, GR203040, GR205171, and RP67580 to antagonize motion-induced emesis in this species (Gardner et al., 1995, 1996; Rudd et al., 1999). It is known that patients with a history of motion sickness also have a higher incidence of chemotherapy-induced emesis (e.g., Morrow, 1984; Shih et al., 2009), post-operative nausea and vomiting, and pregnancy sickness (Bouganim et al., 2012; Warr, 2014). Therefore, it may be expected that netupitant would be useful to control emesis in such patients, where pathways involving motion sickness have been stimulated or perturbed.

## Conclusion

The present studies revealed that netupitant has a broad inhibitory profile to inhibit emesis both in ferrets and *S. murinus*. In particular, a single dose of netupitant at 3 mg/kg given 3 h prior to the administration of cisplatin, provided almost complete protection from acute and delayed emesis; a lower dose of 1 mg/kg, in combination with a single oral dose of palonosetron at 0.1 mg/kg, in combination with daily administrations of dexamethasone (1 mg/kg, i.p.), was also highly effective to reduce acute and delayed emesis, being relatively comparable to a more frequent dosing regimen of ondansetron plus aprepitant in combination with dexamethasone. The convenience of oral dosing, efficacy, and long duration of action are consistent with clinical data. This has been realized by the successful formulation and use of palonosetron plus netupitant in a single pill (Akynzeo^®^) for the treatment of chemotherapy-induced acute and delayed nausea and emesis (Thompson, 2014; Lorusso et al., 2015; Navari, 2015).

## Author Contributions

JR, GH, CG, EL, and CP conceived and designed the experiments. JR, GH, MN, and ZL performed the experiments and data analysis. All authors contributed equally to writing the manuscript.

## Conflict of Interest Statement

The authors declare that the research was conducted in the absence of any commercial or financial relationships that could be construed as a potential conflict of interest.

## References

[B1] Aapro M. Rugo H. Rossi G. Rizzi G. Borroni M. E. Bondarenko I. (2014). A randomized phase III study evaluating the efficacy and safety of NEPA, a fixed-dose combination of netupitant and palonosetron, for prevention of chemotherapy-induced nausea and vomiting following moderately emetogenic chemotherapy. *Ann. Oncol.* 25 1328–1333. 10.1093/annonc/mdu101 24603643 PMC4071754

[B2] Andrews P. L. R. Rudd J. A. (2004). “The role of tachykinins and the tachykinin receptor in nausea and emesis,” in *Handbook of Experimental Pharmacology*, ed. Holzer P. (Berlin: Springer-Verlag), 359–440.

[B3] Andrews P. L. R. Rudd J. A. (2015). “The physiology and pharmacology of nausea and vomiting induced by anti-cancer chemotherapy in humans,” in *Management of Chemotherapy-Induced Nausea and Vomiting: New Agents and New Uses of Current Agents*, ed. Navari R. (London: Springer Health Care Publishers).

[B4] Aranda Aguilar E. Constenla Figueiras M. Cortes-Funes H. Diaz-Rubio Garcia E. Gascon Vilaplana P. Guillem V. (2005). Clinical practice guidelines on antiemetics in oncology. *Expert Rev. Anticancer Ther.* 5 963–972. 10.1586/14737140.5.6.963 16336087

[B5] Ariumi H. Saito R. Nago S. Hyakusoku M. Takano Y. Kamiya H. (2000). The role of tachykinin NK-1 receptors in the area postrema of ferrets in emesis. *Neurosci. Lett.* 286 123–126. 10.1016/S0304-3940(00)01113-7 10825652

[B6] Barnes N. M. Bunce K. T. Naylor R. J. Rudd J. A. (1991). The actions of fentanyl to inhibit drug-induced emesis. *Neuropharmacology* 30 1073–1083. 10.1016/0028-3908(91)90136-Y 1661861

[B7] Bayo J. Fonseca P. J. Hernando S. Servitja S. Calvo A. Falagan S. (2012). Chemotherapy-induced nausea and vomiting: pathophysiology and therapeutic principles. *Clin. Transl. Oncol.* 14 413–422. 10.1007/s12094-012-0818-y 22634529

[B8] Bouganim N. Dranitsaris G. Hopkins S. Vandermeer L. Godbout L. Dent S. (2012). Prospective validation of risk prediction indexes for acute and delayed chemotherapy-induced nausea and vomiting. *Curr. Oncol.* 19 e414–e421. 10.3747/co.19.1074 23300365 PMC3503672

[B9] Bountra C. Bunce K. Dale T. Gardner C. Jordan C. Twissell D. (1993). Anti-emetic profile of a non-peptide neurokinin NK1 receptor. *Eur. J. Pharmacol.* 249 R3–R4. 10.1016/0014-2999(93)90673-6 7506663

[B10] Bubalo J. S. Cherala G. McCune J. S. Munar M. Y. Tse S. Maziarz R. (2012). Aprepitant pharmacokinetics and assessing the impact of aprepitant on cyclophosphamide metabolism in cancer patients undergoing hematopoietic stem cell transplantation. *J. Clin. Pharmacol.* 52 586–594. 10.1177/0091270011398243 21415280

[B11] Chan S. W. Rudd J. A. Lin G. Li P. (2007). Action of anti-tussive drugs on the emetic reflex of *Suncus murinus* (house musk shrew). *Eur. J. Pharmacol.* 559 196–201. 10.1016/j.ejphar.2006.12.008 17254564

[B12] Darmani N. A. (1998). Serotonin 5-HT3 receptor antagonists prevent cisplatin-induced emesis in *Cryptotis parva*: a new experimental model of emesis. *J. Neural Transm.* 105 1143–1154. 10.1007/s007020050118 9928884

[B13] Darmani N. A. Wang Y. Abad J. Ray A. P. Thrush G. R. Ramirez J. (2008). Utilization of the least shrew as a rapid and selective screening model for the antiemetic potential and brain penetration of substance P and NK1 receptor antagonists. *Brain Res.* 1214 58–72. 10.1016/j.brainres.2008.03.077 18471804 PMC2486262

[B14] Darmani N. A. Zhong W. Chebolu S. Mercadante F. (2015). Differential and additive suppressive effects of 5-HT3 (palonosetron)- and NK1 (netupitant)-receptor antagonists on cisplatin-induced vomiting and ERK1/2, PKA and PKC activation. *Pharmacol. Biochem. Behav.* 131 104–111. 10.1016/j.pbb.2015.02.010 25687374

[B15] Darmani N. A. Zhong W. Chebolu S. Vaezi M. Alkam T. (2014). Broad-spectrum antiemetic potential of the L-type calcium channel antagonist nifedipine and evidence for its additive antiemetic interaction with the 5-HT(3) receptor antagonist palonosetron in the least shrew (*Cryptotis parva*). *Eur. J. Pharmacol.* 722 2–12. 10.1016/j.ejphar.2013.08.052 24513517

[B16] De Leon A. (2006). Palonosetron (Aloxi): a second-generation 5-HT(3) receptor antagonist for chemotherapy-induced nausea and vomiting. *Proc. (Bayl. Univ. Med. Cent.)* 19 413–416. 17106506 10.1080/08998280.2006.11928210 PMC1618755

[B17] Eglen R. M. Lee C. H. Smith W. L. Johnson L. G. Clark R. Whiting R. L. (1995). Pharmacological characterization of RS 25259-197, a novel and selective 5-HT3 receptor antagonist, in vivo. *Br. J. Pharmacol.* 114 860–866. 10.1111/j.1476-5381.1995.tb13283.x 7773547 PMC1510198

[B18] Fukuda H. Nakamura E. Koga T. Furukawa N. Shiroshita Y. (1999). The site of the anti-emetic action of tachykinin NK1 receptor antagonists may exist in the medullary area adjacent to the semicompact part of the nucleus ambiguus. *Brain Res.* 818 439–449. 10.1016/S0006-8993(98)01324-9 10082830

[B19] Gardner C. J. Armour D. R. Beattie D. T. Gale J. D. Hawcock A. B. Kilpatrick G. J. (1996). GR205171: a novel antagonist with high affinity for the tachykinin NK1 receptor, and potent broad-spectrum anti-emetic activity. *Regul. Pept.* 65 45–53. 10.1016/0167-0115(96)00071-7 8876035

[B20] Gardner C. J. Twissell D. J. Dale T. J. Gale J. D. Jordan C. C. Kilpatrick G. J. (1995). The broad-spectrum anti-emetic activity of the novel non-peptide tachykinin NK1 receptor antagonist GR203040. *Br. J. Pharmacol.* 116 3158–3163. 10.1111/j.1476-5381.1995.tb15118.x 8719790 PMC1909155

[B21] Geling O. Eichler H. G. (2005). Should 5-hydroxytryptamine-3 receptor antagonists be administered beyond 24 hours after chemotherapy to prevent delayed emesis? Systematic re-evaluation of clinical evidence and drug cost implications. *J. Clin. Oncol.* 23 1289–1294. 10.1200/JCO.2005.04.022 15718327

[B22] Gralla R. J. Bosnjak S. M. Hontsa A. Balser C. Rizzi G. Rossi G. (2014). A phase III study evaluating the safety and efficacy of NEPA, a fixed-dose combination of netupitant and palonosetron, for prevention of chemotherapy-induced nausea and vomiting over repeated cycles of chemotherapy. *Ann. Oncol.* 25 1333–1339. 10.1093/annonc/mdu096 24631949 PMC4071753

[B23] Grunberg S. M. Koeller J. M. (2003). Palonosetron: a unique 5-HT3-receptor antagonist for the prevention of chemotherapy-induced emesis. *Expert Opin. Pharmacother.* 4 2297–2303. 10.1517/14656566.4.12.2297 14640928

[B24] Hasegawa M. Sasaki T. Sadakane K. Tabuchi M. Takeda Y. Kimura M. (2002). Studies for the emetic mechanisms of ipecac syrup (TJN-119) and its active components in ferrets: involvement of 5-hydroxytryptamine receptors. *Jpn. J. Pharmacol.* 89 113–119. 10.1254/jjp.89.113 12120752

[B25] Hesketh P. J. (2008). Chemotherapy-induced nausea and vomiting. *N. Engl. J. Med.* 358 2482–2494. 10.1056/NEJMra0706547 18525044

[B26] Hesketh P. J. Harvey W. H. Harker W. G. Beck T. M. Ryan T. Bricker L. J. (1994). A randomized, double-blind comparison of intravenous ondansetron alone and in combination with intravenous dexamethasone in the prevention of high-dose cisplatin-induced emesis. *J. Clin. Oncol.* 12 596–600. 8120559 10.1200/JCO.1994.12.3.596

[B27] Hesketh P. J. Rossi G. Rizzi G. Palmas M. Alyasova A. Bondarenko I. (2014). Efficacy and safety of NEPA, an oral combination of netupitant and palonosetron, for prevention of chemotherapy-induced nausea and vomiting following highly emetogenic chemotherapy: a randomized dose-ranging pivotal study. *Ann. Oncol.* 25 1340–1346. 10.1093/annonc/mdu110 24608196 PMC4071755

[B28] Huskey S. E. Dean B. J. Bakhtiar R. Sanchez R. I. Tattersall F. D. Rycroft W. (2003). Brain penetration of aprepitant, a substance P receptor antagonist, in ferrets. *Drug Metab. Dispos.* 31 785–791. 10.1124/dmd.31.6.785 12756213

[B29] Ioannidis J. P. Hesketh P. J. Lau J. (2000). Contribution of dexamethasone to control of chemotherapy-induced nausea and vomiting: a meta-analysis of randomized evidence. *J. Clin. Oncol.* 18 3409–3422. 11013282 10.1200/JCO.2000.18.19.3409

[B30] Ito H. Nishibayashi M. Kawabata K. Maeda S. Seki M. Ebukuro S. (2003). Induction of Fos protein in neurons in the medulla oblongata after motion- and X-irradiation-induced emesis in musk shrews (*Suncus murinus*). *Auton. Neurosci.* 107 1–8. 10.1016/S1566-0702(03)00026-2 12927221

[B31] Jordan K. Kasper C. Schmoll H. J. (2005). Chemotherapy-induced nausea and vomiting: current and new standards in the antiemetic prophylaxis and treatment. *Eur. J. Cancer* 41 199–205. 10.1016/j.ejca.2004.09.026 15661543

[B32] Kan K. K. Rudd J. A. Wai M. K. (2006). Differential action of anti-emetic drugs on defecation and emesis induced by prostaglandin E2 in the ferret. *Eur. J. Pharmacol.* 544 153–159. 10.1016/j.ejphar.2006.06.034 16844111

[B33] Lau A. H. Ngan M. P. Rudd J. A. Yew D. T. (2005). Differential action of domperidone to modify emesis and behaviour induced by apomorphine in the ferret. *Eur. J. Pharmacol.* 516 247–252. 10.1016/j.ejphar.2005.05.028 15963978

[B34] Lorusso V. Karthaus M. Aapro M. (2015). Review of oral fixed-dose combination netupitant and palonosetron (NEPA) for the treatment of chemotherapy-induced nausea and vomiting. *Future Oncol.* 11 565–577. 10.2217/fon.14.260 25360998

[B35] Lucot J. B. Obach R. S. McLean S. Watson J. W. (1997). The effect of CP-99994 on the responses to provocative motion in the cat. *Br. J. Pharmacol.* 120 116–120. 10.1038/sj.bjp.0700888 9117085 PMC1564356

[B36] Makale M. T. King G. L. (1992). Surgical and pharmacological dissociation of cardiovascular and emetic responses to intragastric CuSO4. *Am. J. Physiol.* 263 R284–R291. 1354943 10.1152/ajpregu.1992.263.2.R284

[B37] Minami M. Endo T. Hirafuji M. Hamaue N. Liu Y. Hiroshige T. (2003). Pharmacological aspects of anticancer drug-induced emesis with emphasis on serotonin release and vagal nerve activity. *Pharmacol. Ther.* 99 149–165. 10.1016/S0163-7258(03)00057-3 12888110

[B38] Minami M. Endo T. Kikuchi K. Ihira E. Hirafuji M. Hamaue N. (1998). Antiemetic effects of sendide, a peptide tachykinin NK1 receptor antagonist, in the ferret. *Eur. J. Pharmacol.* 363 49–55. 10.1016/S0014-2999(98)00784-5 9877081

[B39] Minami M. Endo T. Monma Y. Shiroshita Y. (1991). Phamracology of emesis induced by ant-cancer drugs. *J. Toxicol. Sci.* 16 35–39. 10.2131/jts.16.SupplementII_35

[B40] Morrow G. R. (1984). Susceptibility to motion sickness and chemotherapy-induced side-effects. *Lancet* 1 390–391. 10.1016/S0140-6736(84)90436-7 6141444

[B41] Navari R. M. (2004). Role of neurokinin-1 receptor antagonists in chemotherapy-induced emesis: summary of clinical trials. *Cancer Invest.* 22 569–576. 10.1081/CNV-200027137 15565815

[B42] Navari R. M. (2009). Pharmacological management of chemotherapy-induced nausea and vomiting: focus on recent developments. *Drugs* 69 515–533. 10.2165/00003495-200969050-00002 19368415

[B43] Navari R. M. (2015). Profile of netupitant/palonosetron (NEPA) fixed dose combination and its potential in the treatment of chemotherapy-induced nausea and vomiting (CINV). *Drug Des. Devel. Ther.* 9 155–161. 10.2147/DDDT.S76158 PMC4277122 25552904

[B44] Naylor R. J. Rudd J. A. (1996). Mechanisms of chemotherapy/radiotherapy-induced emesis in animal models. *Oncology* 53(Suppl. 1), 8–17. 10.1159/000227634 8692557

[B45] Percie du Sert N. Rudd J. A. Apfel C. C. Andrews P. L. (2011). Cisplatin-induced emesis: systematic review and meta-analysis of the ferret model and the effects of 5-HT(3) receptor antagonists. *Cancer Chemother. Pharmacol.* 67 667–686. 10.1007/s00280-010-1339-4 20509026 PMC3043247

[B46] Percie du Sert N. Rudd J. A. Moss R. Andrews P. L. (2009). The delayed phase of cisplatin-induced emesis is mediated by the area postrema and not the abdominal visceral innervation in the ferret. *Neurosci. Lett.* 465 16–20. 10.1016/j.neulet.2009.08.075 19733218

[B47] Ray A. P. Chebolu S. Ramirez J. Darmani N. A. (2009). Ablation of least shrew central neurokinin NK1 receptors reduces GR73632-induced vomiting. *Behav. Neurosci.* 123 701–706. 10.1037/a0015733 19485577 PMC2714262

[B48] Reddy G. K. Gralla R. J. Hesketh P. J. (2006). Novel neurokinin-1 antagonists as antiemetics for the treatment of chemotherapy-induced emesis. *Support. Cancer Ther.* 3 140–142. 10.3816/SCT.2006.n.011 18632487

[B49] Rizzi A. Campi B. Camarda V. Molinari S. Cantoreggi S. Regoli D. (2012). In vitro and in vivo pharmacological characterization of the novel NK(1) receptor selective antagonist Netupitant. *Peptides* 37 86–97. 10.1016/j.peptides.2012.06.010 22732666

[B50] Roila F. Herrstedt J. Aapro M. Gralla R. J. Einhorn L. H. Ballatori E. (2010). Guideline update for MASCC and ESMO in the prevention of chemotherapy- and radiotherapy-induced nausea and vomiting: results of the Perugia consensus conference. *Ann. Oncol.* 21(Suppl. 5), v232–v243. 10.1093/annonc/mdq194 20555089

[B51] Rojas C. Li Y. Zhang J. Stathis M. Alt J. Thomas A. G. (2010a). The antiemetic 5-HT3 receptor antagonist Palonosetron inhibits substance P-mediated responses in vitro and in vivo. *J. Pharmacol. Exp. Ther.* 335 362–368. 10.1124/jpet.110.166181 20724484 PMC3202469

[B52] Rojas C. Thomas A. G. Alt J. Stathis M. Zhang J. Rubenstein E. B. (2010b). Palonosetron triggers 5-HT(3) receptor internalization and causes prolonged inhibition of receptor function. *Eur. J. Pharmacol.* 626 193–199. 10.1016/j.ejphar.2009.10.002 19836386

[B53] Rojas C. Raje M. Tsukamoto T. Slusher B. S. (2014). Molecular mechanisms of 5-HT(3) and NK(1) receptor antagonists in prevention of emesis. *Eur. J. Pharmacol.* 722 26–37. 10.1016/j.ejphar.2013.08.049 24184669

[B54] Rojas C. Stathis M. Thomas A. G. Massuda E. B. Alt J. Zhang J. (2008). Palonosetron exhibits unique molecular interactions with the 5-HT3 receptor. *Anesth. Analg.* 107 469–478. 10.1213/ane.0b013e318172fa74 18633025

[B55] Rubenstein E. B. (2004). Palonosetron: a unique 5-HT(3) receptor antagonist indicated for the prevention of acute and delayed chemotherapy-induced nausea and vomiting. *Clin. Adv. Hematol. Oncol.* 2 284–288. 16163194

[B56] Rudd J. A. Andrews P. L. R. (2004). “Mechanisms of acute, delayed and anticipatory vomiting in cancer and cancer treatment,” in *Management of Nausea and Vomiting in Cancer and Cancer Treatment*, ed. Hesketh P. (New York, NY: Jones and Barlett Publishers Inc.), 15–66.

[B57] Rudd J. A. Jordan C. C. Naylor R. J. (1994). Profiles of emetic action of cisplatin in the ferret: a potential model of acute and delayed emesis. *Eur. J. Pharmacol.* 262 R1–R2. 10.1016/0014-2999(94)90048-5 7813558

[B58] Rudd J. A. Jordan C. C. Naylor R. J. (1996). The action of the NK1 tachykinin receptor antagonist, CP 99,994, in antagonizing the acute and delayed emesis induced by cisplatin in the ferret. *Br. J. Pharmacol.* 119 931–936. 10.1111/j.1476-5381.1996.tb15761.x 8922742 PMC1915933

[B59] Rudd J. A. Naylor R. J. (1994). Effects of 5-HT3 receptor antagonists on models of acute and delayed emesis induced by cisplatin in the ferret. *Neuropharmacology* 33 1607–1608. 10.1016/0028-3908(94)90136-8 7760983

[B60] Rudd J. A. Naylor R. J. (1995). “Opioid receptor involvement in emesis and anti-emesis,” in *Serotonin and the Scientific Basis of Anti-Emetic Therapy*, eds Andrew P. L. R. Reynolds D. J. M. Davis C. J. (London: Oxford Clincal Communications), 208–219.

[B61] Rudd J. A. Naylor R. J. (1996). An interaction of ondansetron and dexamethasone antagonizing cisplatin-induced acute and delayed emesis in the ferret. *Br. J. Pharmacol.* 118 209–214. 10.1111/j.1476-5381.1996.tb15388.x 8735616 PMC1909635

[B62] Rudd J. A. Naylor R. J. (1997). The actions of ondansetron and dexamethasone to antagonise cisplatin-induced emesis in the ferret. *Eur. J. Pharmacol.* 322 79–82. 10.1016/S0014-2999(97)00073-3 9088874

[B63] Rudd J. A. Ngan M. P. Wai M. K. (1999). Inhibition of emesis by tachykinin NK1 receptor antagonists in *Suncus murinus* (house musk shrew). *Eur. J. Pharmacol.* 366 243–252. 10.1016/S0014-2999(98)00920-0 10082206

[B64] Rupniak N. M. Tattersall F. D. Williams A. R. Rycroft W. Carlson E. J. Cascieri M. A. (1997). In vitro and in vivo predictors of the anti-emetic activity of tachykinin NK1 receptor antagonists. *Eur. J. Pharmacol.* 326 201–209. 10.1016/S0014-2999(97)85415-5 9196273

[B65] Saito M. Aogi K. Sekine I. Yoshizawa H. Yanagita Y. Sakai H. (2009). Palonosetron plus dexamethasone versus granisetron plus dexamethasone for prevention of nausea and vomiting during chemotherapy: a double-blind, double-dummy, randomised, comparative phase III trial. *Lancet Oncol.* 10 115–124. 10.1016/S1470-2045(08)70313-9 19135415

[B66] Sam T. S. Chan S. W. Rudd J. A. Yeung J. H. (2001). Action of glucocorticoids to antagonise cisplatin-induced acute and delayed emesis in the ferret. *Eur. J. Pharmacol.* 417 231–237. 10.1016/S0014-2999(01)00915-3 11334855

[B67] Shih V. Wan H. S. Chan A. (2009). Clinical predictors of chemotherapy-induced nausea and vomiting in breast cancer patients receiving adjuvant doxorubicin and cyclophosphamide. *Ann. Pharmacother.* 43 444–452. 10.1345/aph.1L437 19193584

[B68] Siddiqui M. A. Scott L. J. (2004). Palonosetron. *Drugs* 64 1125–1132; discussion1133–1124. 10.2165/00003495-200464100-00006 15139789

[B69] Singh L. Field M. J. Hughes J. Kuo B. S. Suman-Chauhan N. Tuladhar B. R. (1997). The tachykinin NK1 receptor antagonist PD 154075 blocks cisplatin-induced delayed emesis in the ferret. *Eur. J. Pharmacol.* 321 209–216. 10.1016/S0014-2999(96)00950-8 9063690

[B70] Stathis M. Pietra C. Rojas C. Slusher B. S. (2012). Inhibition of substance P-mediated responses in NG108-15 cells by netupitant and palonosetron exhibit synergistic effects. *Eur. J. Pharmacol.* 689 25–30. 10.1016/j.ejphar.2012.05.037 22683863

[B71] Stoltz R. Cyong J. C. Shah A. Parisi S. (2004). Pharmacokinetic and safety evaluation of palonosetron, a 5-hydroxytryptamine-3 receptor antagonist, in U.S. and Japanese healthy subjects. *J. Clin. Pharmacol.* 44 520–531. 10.1177/0091270004264641 15102873

[B72] Tattersall F. D. Rycroft W. Cumberbatch M. Mason G. Tye S. Williamson D. J. (2000). The novel NK1 receptor antagonist MK-0869 (L-754,030) and its water soluble phosphoryl prodrug, L-758,298, inhibit acute and delayed cisplatin-induced emesis in ferrets. *Neuropharmacology* 39 652–663. 10.1016/S0028-3908(99)00172-0 10728886

[B73] Tattersall F. D. Rycroft W. Francis B. Pearce D. Merchant K. MacLeod A. M. (1996). Tachykinin NK1 receptor antagonists act centrally to inhibit emesis induced by the chemotherapeutic agent cisplatin in ferrets. *Neuropharmacology* 35 1121–1129. 10.1016/S0028-3908(96)00020-2 9121615

[B74] Tattersall F. D. Rycroft W. Hargreaves R. J. Hill R. G. (1993). The tachykinin NK1 receptor antagonist CP-99,994 attenuates cisplatin induced emesis in the ferret. *Eur. J. Pharmacol.* 250 R5–R6. 10.1016/0014-2999(93)90649-3 8119305

[B75] Tattersall F. D. Rycroft W. Hill R. G. Hargreaves R. J. (1994). Enantioselective inhibition of apomorphine-induced emesis in the ferret by the neurokinin1 receptor antagonist CP-99,994. *Neuropharmacology* 33 259–260. 10.1016/0028-3908(94)90018-3 8035913

[B76] Tattersall F. D. Rycroft W. Marmont N. Cascieri M. Hill R. G. Hargreaves R. J. (1995). Enantiospecific inhibition of emesis induced by nicotine in the house musk shrew (*Suncus murinus*) by the neurokinin1 (NK1) receptor antagonist CP-99,994. *Neuropharmacology* 34 1697–1699. 10.1016/0028-3908(95)00164-6 8788968

[B77] Thompson C. A. (2014). Netupitant-palonosetron combination approved by FDA. *Am. J. Health Syst. Pharm.* 71 2000 10.2146/news140079 25404586

[B78] Tonini G. Vincenzi B. Santini D. (2005). New drugs for chemotherapy-induced nausea and vomiting: focus on palonosetron. *Expert Opin. Drug Metab. Toxicol.* 1 143–149. 10.1517/17425255.1.1.143 16922656

[B79] Tsuchiya M. Fujiwara Y. Kanai Y. Mizutani M. Shimada K. Suga O. (2002). Anti-emetic activity of the novel nonpeptide tachykinin NK1 receptor antagonist ezlopitant (CJ-11,974) against acute and delayed cisplatin-induced emesis in the ferret. *Pharmacology* 66 144–152. 10.1159/000063796 12372904

[B80] Ueno S. Matsuki N. Saito H. (1988). *Suncus murinus* as a new experimental model for motion sickness. *Life Sci.* 43 413–420. 10.1016/0024-3205(88)90520-6 2899827

[B81] Warr D. (2014). Prognostic factors for chemotherapy induced nausea and vomiting. *Eur. J. Pharmacol.* 722 192–196. 10.1016/j.ejphar.2013.10.015 24157977

[B82] Watanabe Y. Okamoto M. Ishii T. Takatsuka S. Taniguchi H. Nagasaki M. (2008). Long-Lasting Anti-emetic Effect of T-2328, a Novel NK(1) Antagonist. *J. Pharmacol. Sci.* 107 151–158. 10.1254/jphs.08027FP 18544900

[B83] Watson J. W. Gonsalves S. F. Fossa A. A. McLean S. Seeger T. Obach S. (1995). The anti-emetic effects of CP-99,994 in the ferret and the dog: role of the NK1 receptor. *Br. J. Pharmacol.* 115 84–94. 10.1111/j.1476-5381.1995.tb16324.x 7544198 PMC1908747

[B84] Wong E. H. Clark R. Leung E. Loury D. Bonhaus D. W. Jakeman L. (1995). The interaction of RS 25259-197, a potent and selective antagonist, with 5-HT3 receptors, in vitro. *Br. J. Pharmacol.* 114 851–859. 10.1111/j.1476-5381.1995.tb13282.x 7773546 PMC1510197

[B85] Zaman S. Woods A. J. Watson J. W. Reynolds D. J. Andrews P. L. (2000). The effect of the NK1 receptor antagonist CP-99,994 on emesis and c-fos protein induction by loperamide in the ferret. *Neuropharmacology* 39 316–323. 10.1016/S0028-3908(99)00113-6 10670427

